# LyeTx I-b Peptide Attenuates Tumor Burden and Metastasis in a Mouse 4T1 Breast Cancer Model

**DOI:** 10.3390/antibiotics10091136

**Published:** 2021-09-21

**Authors:** Mostafa A. L. Abdel-Salam, Bárbara Pinto, Geovanni Cassali, Lilian Bueno, Gabriela Pêgas, Fabrício Oliveira, Irismara Silva, André Klein, Elaine Maria de Souza-Fagundes, Maria Elena de Lima, Juliana Carvalho-Tavares

**Affiliations:** 1 Programa de Pós-Graduação em Ciências Biológicas: Fisiologia e Farmacologia, Departamento de Fisiologia e Biofísica, Instituto de Ciências Biológicas, Universidade Federal de Minas Gerais, Belo Horizonte 31270-901, Brazil; mostafaabdellatif2020@gmail.com (M.A.L.A.-S.); barbara_fernandes_pinto@hotmail.com (B.P.); elainefagundes@ufmg.br (E.M.d.S.-F.); 2 Departamento de Patologia Geral, Instituto de Ciências Biológicas, Universidade Federal de Minas Gerais, Belo Horizonte 31270-901, Brazil; cassalig@icb.ufmg.br (G.C.); gabrielapegas@yahoo.com.br (G.P.); oliveirafms13@gmail.com (F.O.); 3 Departamento de Parasitologia, Instituto de Ciências Biológicas, Universidade Federal de Minas Gerais, Belo Horizonte 31270-901, Brazil; lilacerdabueno@gmail.com; 4 Programa de Pós-Graduação em Ciências Biológicas: Fisiologia e Farmacologia, Departamento de Farmacologia, Instituto de Ciências Biológicas, Universidade Federal de Minas Gerais, Belo Horizonte 31270-901, Brazil; irissousa.s@hotmail.com (I.S.); klein@icb.ufmg.br (A.K.); 5 Programa de Pós-Graduação em Medicina-Biomedicina, Faculdade Santa Casa de Belo Horizonte, Belo Horizonte 30110-005, Brazil

**Keywords:** breast cancer, LyeTx I-b peptide, immunomodulation, leukocyte recruitment, cytokines, anticancer activity of LyeTx I-b, *Lycosa erythrognatha*

## Abstract

Cationic anticancer peptides have exhibited potent anti-proliferative and anti-inflammatory effects in neoplastic illness conditions. LyeTx I-b is a synthetic peptide derived from *Lycosa erythrognatha* spider venom that previously showed antibiotic activity in vitro and in vivo. This study focused on the effects of LyeTxI-b on a 4T1 mouse mammary carcinoma model. Mice with a palpable tumor in the left flank were subcutaneously or intratumorally injected with LyeTx I-b (5 mg/kg), which significantly decreased the tumor volume and metastatic nodules. Histological analyses showed a large necrotic area in treated primary tumors compared to the control. LyeTxI-b reduced tumor growth and lung metastasis in the 4T1 mouse mammary carcinoma model with no signs of toxicity in healthy or cancerous mice. The mechanism of action of LyeTx I-b on the 4T1 mouse mammary carcinoma model was evaluated in vitro and is associated with induction of apoptosis and cell proliferation inhibition. Furthermore, LyeTx I-b seems to be an efficient regulator of the 4T1 tumor microenvironment by modulating several cytokines, such as TGF-β, TNF-α, IL-1β, IL-6, and IL-10, in primary tumor and lung, spleen, and brain. LyeTx I-b also plays a role in leukocytes rolling and adhesion into spinal cord microcirculation and in the number of circulating leukocytes. These data suggest a potent antineoplastic efficacy ofLyeTx I-b.

## 1. Introduction

Breast cancer is the most common cancer in women and the second leading cause of cancer death worldwide after lung cancer [1,2]. Data from the World Health Organization (WHO) estimated that in 2020 more than 2.3 million women were diagnosed with breast cancer globally, of which 685,000 evolved to death [3]. Moreover, the incidence of the disease is increasing in both developing and developed countries [4]. For instance, it is estimated that 52% of all breast cancer cases occur in low-income and middle-income countries (LIMICs) [5]. Many risk factors are associated with breast cancer development, including lifestyle, age, hormonal status, and family history, influencing disease susceptibility [6,7]. Besides that, breast cancer is highly heterogeneous, making its diagnosis and the implementation of effective therapies difficult [8].

Relevant advances in cancer therapy have been reached, but the challenges continue to exist [9]. Although the currently available breast cancer treatments can delay the tumor evolution, the prognosis remains poor [10]. Moreover, these therapeutic strategies (e.g., surgery, chemotherapy, radiotherapy, and biological and hormonal therapies) present diverse side effects, including fatigue, pain, nausea, hair loss, diarrhea, mucosal inflammation, weakness, thrombosis, neuropathy, cardiotoxicity, and renal oxidative stress [11,12]. Therefore, new therapeutic targets are needed to reduce cancer progression and improve patient outcomes [10].

Metastasis and drug resistance are still a global problem in patients with advanced breast cancer [10,13]. Breast cancer cells can migrate from the primary tumor to distant organs, such as the brain, bones, lung, and liver, through the lymphatic and circulatory systems, causing metastasis, which is recognized as the stage IV of cancer progression [14,15]. The 4T1 mouse mammary tumor cells line has been used to study spontaneous breast cancer and tumor metastasis. This model presents kinetics similar to stage IV human breast cancer, exhibiting aggressive cancer cell growth followed by leukocytosis [16,17,18].

The immune ecosystem of the tumor plays an essential role in the development and progression of breast cancer [19]. Moreover, it is well known that the inflammatory process in a tumor microenvironment leads to leukocyte infiltration and an increase in interleukins, fibroblast, and growth factors secretion [20]. In addition, tumor-infiltrating lymphocytes have been associated with the increase of cancer survival and metastasis [21].

The human innate immune system contains many natural antimicrobial peptides (AMPs) for fighting against infections and cancer, among other diseases. AMPs are also widely found in nature like animal venoms and plants [22] and a peptide database from Nebraska University (aps.unmc.edu, accessed on: 15 September 2021) has cataloged 3273 peptides to date.

Peptide-based drug delivery is one of the best strategies to overcome drug failure during conventional therapy. Until recently, over 150 peptides have entered in clinical studies [23] and this number has been increased. Moreover, the therapeutic peptides have shown signs of future success to treat cancer diseases due to advantages, such as affinity to bind with receptors, selectivity, and specificity to tumor cells, with low toxicity in normal tissues [24]. Cationic antimicrobial peptides could induce a rapid cell membrane disruption effect, and thus might be reducing drug resistance in cancer cells [25]. In some cases, the cancer tissue is more susceptible to the action of these peptides, as for example, the higher number of microvilli in cancer cells than normal cells, increases the possibility of interaction with cationic antimicrobial peptides [26]. 

AMPs have been known, specially to inhibit or kill several pathogenic microorganisms [27,28,29]. The interest in AMPs as novel biologic and immunotherapeutic agents against human malignancies has increased significantly [30]. For example, several AMPs have exhibited a potent cytotoxic effect and selectivity against many types of malignant tumors, such as carcinoma, adenoma, sarcoma, and leukemia [22]. Then, AMPs could be an interesting target for the treatment of cancer since they are considered cheap, well-tolerated, and effective sources of drugs for the first-line treatment of cancer [31,32]. Various therapeutic peptides alone or in combination with other conventional chemotherapeutic agents have shown high tolerance and efficacy in cancer clinical trials [33]. In this context, animal venoms are considered rich sources of AMPs [29,34,35].

LyeTxI, a peptide with 25 amino acid residues, was purified from the venom of the spider *L. erythrognatha* and has exhibited antimicrobial effects against bacteria and fungi [36]. LyeTx I-b is a synthetic peptide differing from LyeTx I by lacking a cysteine residue at position 16. LyeTx I-b was active against diverse bacteria in in vitro and in vivo assays [35,36,37] and presented a higher affinity in phospholipid membrane assays compared to LyeTx I [38]. Furthermore, a previous study conducted by our group showed that intraocular injection of LyeTx I-b suppressed neovascularization in ocular disease using a chorioallantoic membrane model [39]. LyeTxI-b also showed antibacterial activity against resistant keratitis caused by ocular infection with no sign of toxicity [40]. Moreover, this peptide presented cytotoxicity against U87-MGglioblastoma cells involving necroptosis, which was suggested as its principal mechanism of action in these cells [41]. It also caused mild cytotoxicity against normal fibroblasts of human and monkey cell lines and low hemolytic activity in human erythrocytes.

In this context, this study aimed to investigate the potential in vivo antitumor activity of LyeTx I-b, focusing on immunomodulatory effects in primary tumor, spleen, brain, and lung in the 4T1 murine metastatic model.

## 2. Results

### 2.1. LyeTx I-b Peptide Shows Cytotoxicity against Human and Mouse Breast Cancer Cells

In order to evaluate the anticancer potential of LyeTxI-b in vivo, we first investigated its cytotoxicity against different lineages of human and mouse breast cancer cells. The MTT assay was used to evaluate cell viability/proliferation. LyeTxI-b exhibited a significant cytotoxic effect against breast carcinoma cells at a lower micromolar range (<10 µM) compared to carboplatin, a standard drug used in the clinical context (Table 1). LyeTxI-b presented IC_50_ values in the same order of magnitude (~5 to 7 µM) for the different tumor cell lines employed. Representative concentration-response curves are displayed along with Table 1. Interestingly, this peptide was more cytotoxic than carboplatin (IC_50_ > 100 µM to MCF-7 and 4T1; and 69.20 ± 1.22 µM to MDA-MB-231 µM) against all lineages, mainly against the triple-negative breast cancer lineage (MDA-MB-231 and 4T1).

### 2.2. LyeTxI-b Peptide Reduces the Clonogenic Survival of 4T1 Cells

**Figure 1:**
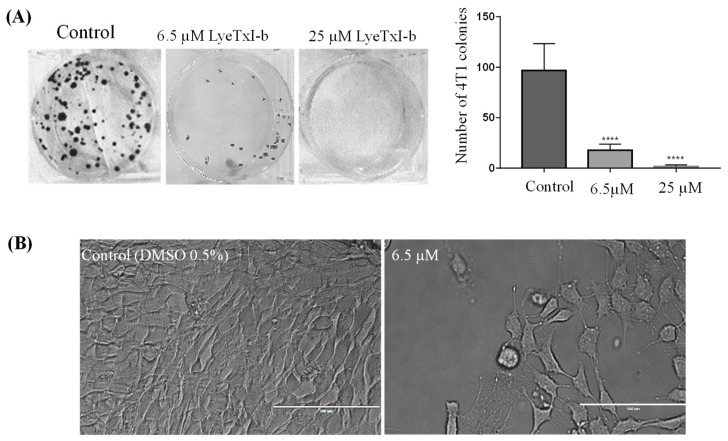
Quantification of the survival of 4T1 single cell colonies after the treatment with LyeTx I-b (IC_50_ and IC_80_). The IC_50_ and IC_80_ were determined by the MTT method. Representative images of colonies of 4T1 after 10 days (**A**) and morphology of untreated (control) and treated cells with IC_50_ concentration of peptide (**B**) are shown. Data represent the mean ± SD of duplicates of three independent experiments, One-way ANOVA followed by Dunnett’s comparison test, **** *p* < 0.0001 compared to the control group.

The efficacy of LyeTx I-b was evaluated in vitro using murine breast lineage 4T1 since this cell line was chosen to investigate the anticancer potential in vivo of this peptide. Clonogenic assays have been used in drug discovery programs as a predictive model to evaluate the in vivo efficacy of anticancer compounds [42]. LyeTxI-b reduced the clonogenic survival of 4T1 cells, which can be observed by a decrease in the number of colonies compared to control (Figure 1A, colonies images). LyeTx I-b prevented the proliferation of clonogenic cells in vitro in a dose-dependent manner, suggesting that this peptide has tumoricidal activity (Figure 1A). Figure 1B displays representative images of 4T1 cells untreated (DMSO, 0.5%) with typical morphology. After treatment with 6.5 µM (IC_50_ value) of peptide, it was observed that LyeTxI-b induced alteration of shape, size, and amount of cells.

### 2.3. LyeTxI-b Increases Sub-Diploid DNA Content and Exposure to Phosphatidylserine

**Figure 2:**
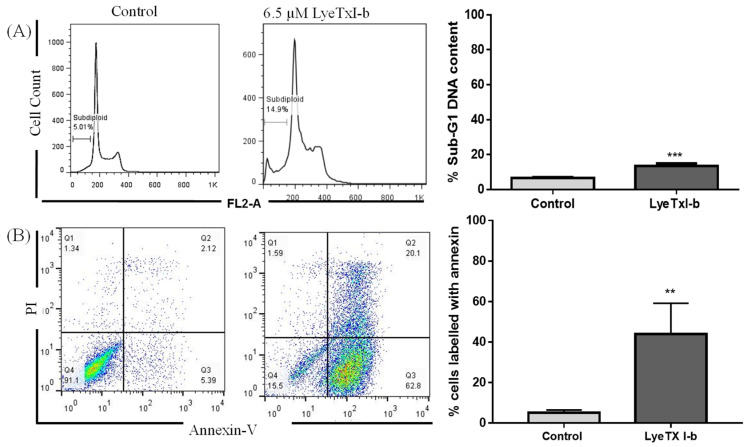
(**A**) Representative histograms of cell cycle analysis after exposure to LyeTx I-b and carboplatin at IC_50_ values for 48 h. The results represent three independent experiments in triplicate and show significant differences between the negative control and the treated cells in terms of the G0/G1 (sub-diploid) population. (**B**) Illustrative histogram representing the quantification of the proportions of the dead and live cells after exposure of 4T1 cells to 6.5 µM of LyeTx I-b for 24 h and staining with annexin V/Propidium iodide (PI). The number of cells in each quadrant (Q) gate can be quantified as follows: Q4 = live cells (PI negative/annexin V negative); Q3 = apoptotic cells (PI negative/annexin V positive); Q2 = necrotic or late apoptotic cells (PI positive/annexin V positive); and, eventually, Q1 = nuclei without plasma membrane = (PI positive/annexin V negative). Three independent experiments in triplicate and unpaired *t*-test were performed, ** *p* < 0.01; *** *p* < 0.001 compared to the control group.

Apoptosis evasion is one of the most important hallmarks of cancer. As more apoptosis-inducing anticancer drugs are discovered, the most effective treatment could emerge. In this context, the sub-diploid DNA content was determined to evaluate the in vitro potential of the peptide LyeTx I-b to induce apoptosis in 4T1 cells. Cells undergoing apoptotic cell death display an increase in sub-diploid DNA content that can be correlated with DNA fragmentation and phosphatidylserine exposure. Both cell alterations were measured by flow cytometry after 48 h treatment of 4T1 cells with LyeTxI-b at the IC_50_ concentration. A significant increase in the sub-diploid (sub-G1) DNA content was observed in the LyeTxI-b -treated cells (Figure 2A) compared to the control (PBS), suggesting induction of apoptosis or necrosis. To distinguish between apoptosis and necrosis, the evaluation of phosphatidylserine exposure, an early event of apoptosis associated with the phosphatidylserine translocation from the internal to the external leaf of membrane, was investigated by labeling cells with annexin-V and propidium iodide (PI). The 4T1 cells were treated with 6.5 µM of LyeTxI-b for 24 h and stained with annexin V/PI for 15 min. The flow cytometry data (Figure 2B) demonstrated that the majority of the 4T1 population was distributed or located in the areas of annexin positive (i.e., quadrant Q3 represents the early apoptotic area and the minor population was double-positive (PI positive/annexin V positive), representing late apoptosis or necroptosis in the Q2 area). By contrast, all control cells were located in the double negative Q4 area of live cells (PI negative/annexin V-negative). 

### 2.4. Sub-Acute Toxicity of LyeTx I-b Peptide

**Figure 3:**
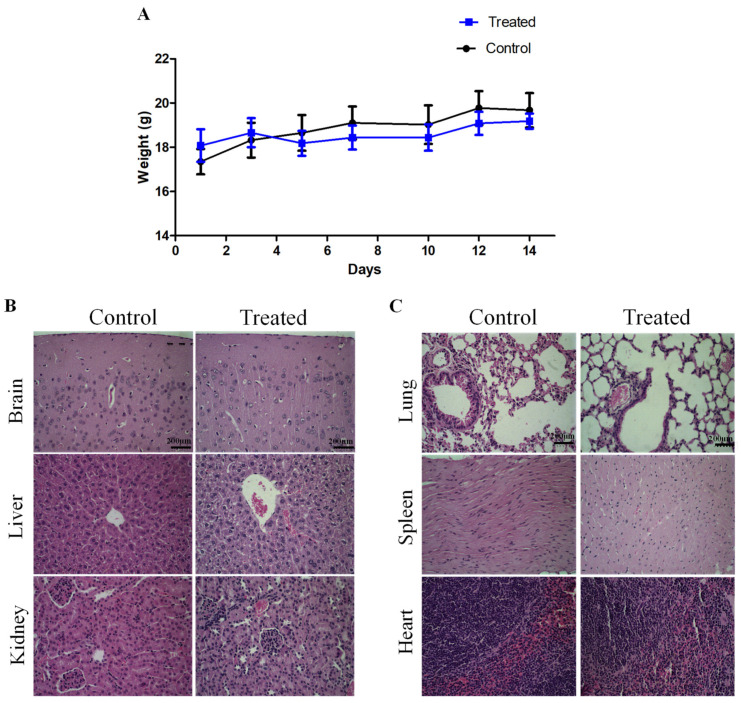
In vivo sub-acute toxicity after i.p. injection of LyeTxI-b peptide (5 mg/kg) (**A**) Effect of the peptide (5 mg/kg) on body weight of BALB/c mice over 14 days, *n* = 6. (**B**) Murine histopathological analyses of brain, liver, and kidney (**C**) Murine histopathological analyses of lung, spleen and heart. All tissues were stained with hematoxylin and eosin. Objective, 40×. Scale bar, 200 μm.

In order to investigate the in vivo sub-acute toxicity of LyeTxI-b, female BALB/c mice received intraperitoneal (i.p.) injection of the peptide (5 mg/kg) (seven doses, 48 h apart). Clinical signs, such as toxicity, skin ulcer, diarrhea, and mortality, were not observed over the period assessed. Body weight of animals treated with saline (control group) or LyeTx I-b dissolved in saline (5 mg/kg) was evaluated over 14 days. No difference was observed in body weight between the control and treated animal groups throughout the evaluation period (Figure 3A). Histopathological and macroscopic analysis (lung, kidney, spleen, heart, brain, and liver) demonstrated that LyeTxI-b did not cause lesions or significant alterations in these organs (Figure 3B,C). In the area of the Bowman’s capsule, glomerulus and tubules from the renal cortex showed normal patterns compared to control. The liver components i.e., parenchyma, hepatocytes, central vein, bile duct morphology displayed normal patterns (Figure 3B). The lung histopathology was also preserved, without changes in alveoli, alveolar air spaces (Figure 3C). The analysis of spleen revealed well preserved white pulp, red pulp, central artery, and capsule. Moreover, the heart muscle fiber, intercalated disc, and nuclei morphology were normal as well (Figure 3C).

Furthermore, the hematological values of the control and treated groups did not show abnormal patterns, which generally include morphology and counts related to age and sex, except for the group treated with the peptide, which displayed a reduction in platelet counts (Table 2). 

### 2.5. Intratumoral Administration of LyeTxI-b Decreases Tumor Growth and Lung Metastatic Nodules

Considering the safety presented by LyeTx I-b in animals and its in vitro cytotoxic activity, we investigated the in vivo anticancer effect of this peptide using the triple-negative breast cancer 4T1 model. As peptides can have short half-lives due to extensive proteolysis in blood, some strategies to overcome this problem include their administration through alternatives routes, such as inhalation, oral, intranasal, and subcutaneous [43]. Two administration routes, intratumoral and subcutaneous, were selected to investigate the antitumor activity of LyeTx I-b. 

**Figure 4:**
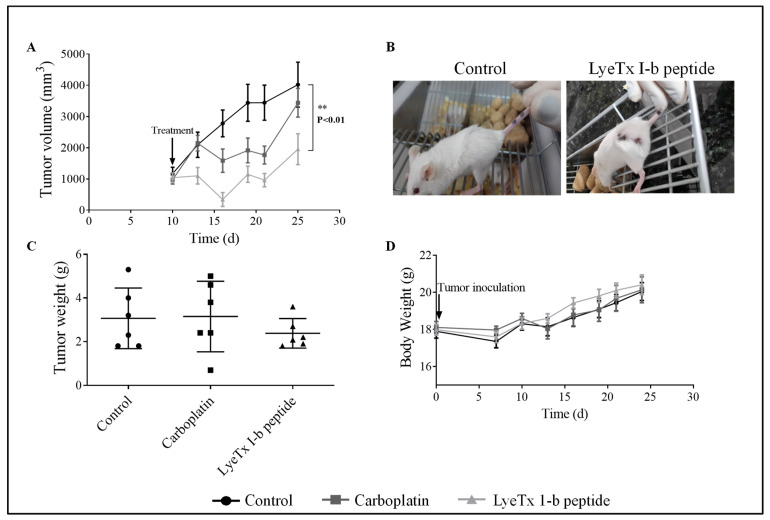
Effects of Tx I-b and carboplatin treatments (5 mg/kg) on 4T1 murine mammary carcinoma on the volume and weight of the tumor, and the body weight, *n* = 6. (**A**) LyeTx I-b decreased the tumor volume compared to the control group. (**B**) Representative images demonstrating a reduction in the size of tumor after treatment with LyeTx I-b. (**C**) LyeTx I-b reduced tumor weight. (**D**) No body weight differences over 25 days post-inoculation were observed on 4T1 murine mammary carcinoma model in the control, LyeTx I-b, and carboplatin groups. Data represent the mean ± SD, One-way ANOVA followed by Tukey’s multiple comparisons test, ** *p* < 0.01.

On day 10 post-tumor inoculation, when the tumor volume reached approximately 1000 mm^3^, animals received intratumoral injections of 100 µL of LyeTx I-b or carboplatin (5 mg/kg) dissolved in saline. At the end of the experiments, internal tumor burden was necropsied, and the enlargement of spleens and lymphnodes was notably observed in the vehicle control (data not shown). Figure 4A shows that LyeTxI-b significantly reduced tumor volume compared to the control group. Representative images of animals of the control group and treated with LyeTx I-b or carboplatin are presented in Figure 4B. No differences in tumor weight or body weight of animals were observed in both groups (peptide or carboplatin) compared to the control (Figure 4C,D, respectively).

**Figure 5:**
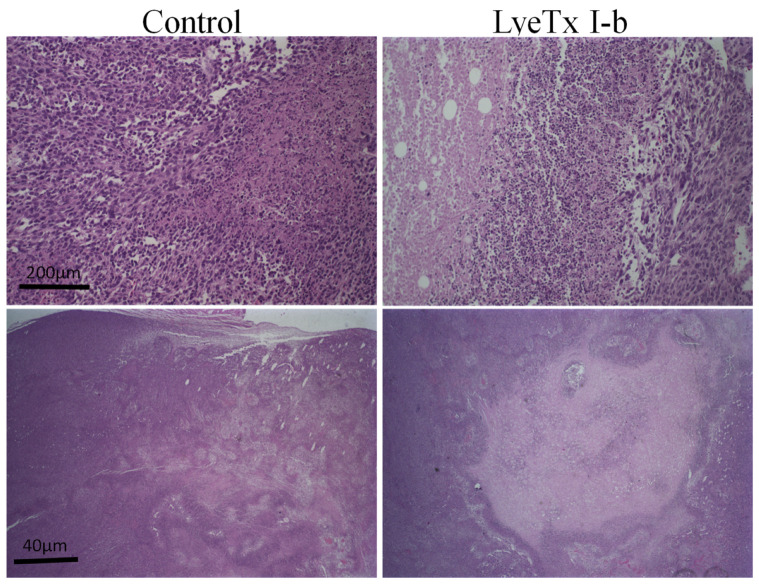
Assessment of morphological aspects of 4T1 murine primary tumor. Representative pictures of hematoxylin and eosin staining using low magnification showed a large necrosis area in the treated group compared to the untreated group, *n* = 6. In the control group, were observed inflammatory cell infiltrate, necrotic area, and viable tumor cells, whereas in the treated group the extensive necrotic area was characterized by the presence of nucleus chromatin condensation accompanied by fragmented nuclei into various segments (karyorrhexis). Objectives: 4× and 20×. Scale bars: 200 µm and 40 µm.

However, differences in metastatic foci in the lungs after the treatment with this peptide could be observed. Qualitative histopathological analysis showed that the primary tumor in the control group presented an increase in infiltrated mononuclear (lymphocytes and macrophages) inflammatory cells associated with the high growth of neoplastic cells and the formation of necrotic areas. An extensive necrotic area in the primary tumor was observed in the group treated with LyeTxI-b, as evidenced by the presence of nucleus chromatin condensation accompanied by the fragmentation of nuclei into various segments (karyorrhexis) (Figure 5).

**Figure 6:**
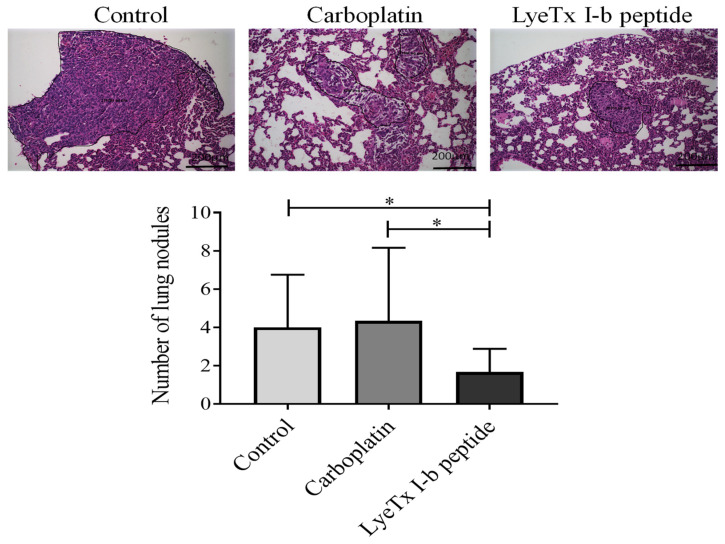
4T1 metastasis in the lung parenchyma. A significant reduction in the number of metastatic lesions in the LyeTx I-b group compared to the carboplatin treated and control groups was observed, *n* = 6. Hematoxylin and eosin (H&E) staining. Data represent the mean ± SD, One-way ANOVA followed by Tukey’s multiple comparisons test, * *p* < 0.05.

The assessment of lung metastasis shows that LyeTx I-b significantly reduced the number of metastatic focus in animals in comparison to carboplatin and control groups (Figure 6). The mean number of metastases for the peptide-treated group was 1.66, 4.33 for carboplatin, and 3.66 for the control group. Moreover, LyeTx I-b reduced the lesion areas (Figure 6).

### 2.6. Systemic Subcutaneous Injection of LyeTx I-b Reduces Tumor Volume

**Figure 7:**
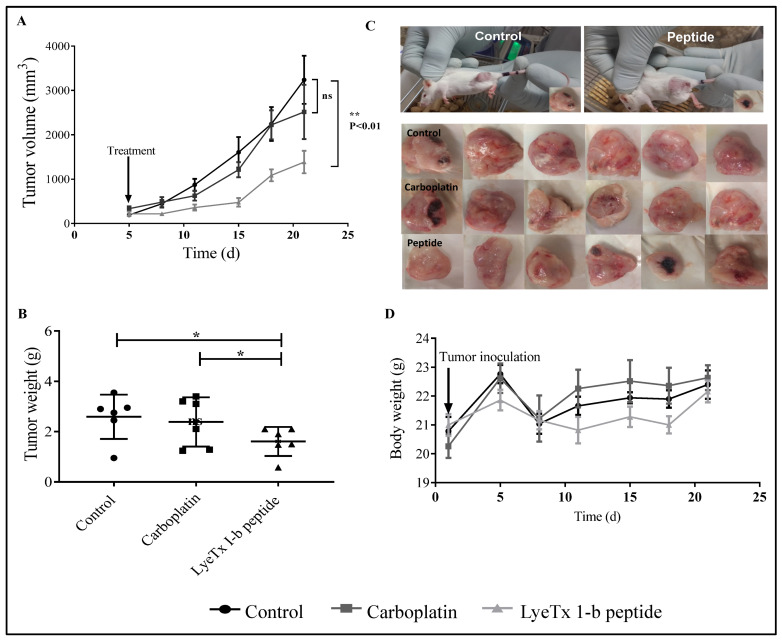
Effects of subcutaneous LyeTx I-b treatment on tumor volume, tumor weight, and body weight after 4T1 murine mammary carcinoma induction, *n* = 6. (**A**) LyeTx I-b (5 mg/kg) significantly decreased the tumor volume compared to the control and carboplatin (5 mg/kg). (**B**) LyeTx I-b reduced the tumor weight compared to the control and carboplatin groups. (**C**) Representative images demonstrating a reduction in the size of the tumor after the treatment with LyeTx I-b. (**D**) Influence of tumor induction and drug treatment on BALB/c body weight over 21 days post-inoculation of 4T1 tumor. Data represent the mean ± SD, One-way ANOVA followed by Tukey’s multiple comparisons test, * *p* < 0.05, ** *p* < 0.01. ns: means not statistically significant.

The subcutaneous administration of LyeTx I-b was used to evaluate the efficacy of the peptide through lymphatic and vascular circulatory systems. On day 5 post-tumor inoculation (tumor volume of approximately 400 mm^3^), animals received subcutaneous injections of LyeTxI-b (100 µL), carboplatin (5 mg/kg), or saline (control group) (5 doses/72 h apart). The results demonstrated that LyeTxI-b significantly reduced the tumor volume (Figure 7A,C) and tumor weight (Figure 7B) compared to the control and carboplatin groups. The mean for the tumor weights of the control and carboplatin groups were 2.92 g and 2.38 g, respectively, while for the peptide-treated group was 1.6 g. Furthermore, the mean for tumor volume of the group treated with LyeTx I-b was 1383 mm^3^, while for the control group was 3236 mm^3^ and 2513 mm^3^ for carboplatin (Figure 7B). No difference was observed in the body weight between the control, LyeTx I-b, and carboplatin groups (Figure 7D).

### 2.7. Systemic Subcutaneous Injection of LyeTx I-b Peptide Reduces Neoplastic Cells in Primary Tumor

**Figure 8:**
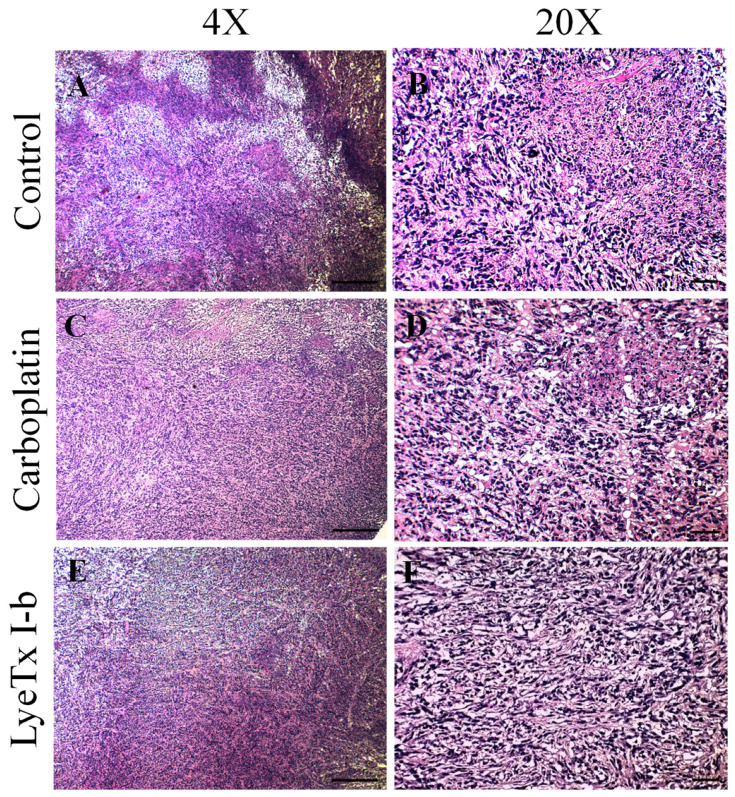
Histological effects of subcutaneous administration of LyeTx I-b on4T1 murine primary tumor, *n* = 6. (**A**,**B**) The control group exhibited the presence of neoplastic cells with a high degree of cellular polymorphism. (**C**,**D**) The carboplatin group showed inflammatory infiltrate characterized by mononuclear and neoplastic cells with a high degree of polymorphisms. (**E**,**F**) The reduction of areas of necrosis and fibrosis in the tumor periphery. (**B**,**F**) The group treated with LyeTx I-b presented a reduction in neoplastic cells and necrotic areas, associated with increased deposition of fibrous connective tissue. H&E staining. Objectives: 4× and 20×. Scale bars: 200 µm and 40 µm.

Histopathological analysis of primary tumor in the control group demonstrated a remarkable neoplastic lesion characterized by increased development of neoplastic cells with a high degree of cellular polymorphism accompanied by large necrotic areas in peripheral regions, moderate presence of fibrosis, and mononuclear inflammatory cells. The carboplatin group showed apparent augmentation of infiltrated inflammatory cells and neoplastic cells with cellular polymorphism. On the other hand, the LyeTx I-b group presented a notable reduction of neoplastic cells and necrotic areas, associated with increased deposition of fibrous connective tissue in the primary tumor (Figure 8).

### 2.8. No Liver Function Alteration Was Induced by LyeTx I-b Treatment

**Figure 9:**
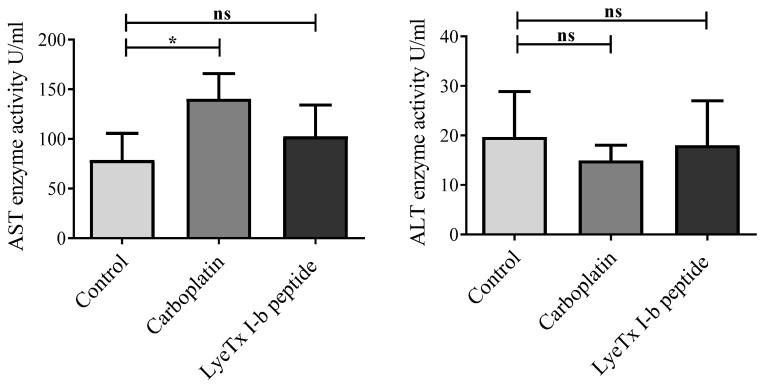
Plasma aspartate aminotransferase (AST) and alanine aminotransferase (ALT) activities in 4T1 tumor-bearing mice. Balb/c mice were injected subcutaneously 5 times, every 72 h, for 15 days with saline (control), LyeTX I-b (5 mg/kg), or carboplatin (5 mg/kg). AST and ALT levels were measured by using the colorimeter testing kit in triplicate, *n* = 6. Data represent the mean ± SD, One-way ANOVA followed by Tukey’s multiple comparisons test, * *p* < 0.05. ns: means not statistically significant.

We further tested whether LyeTx I-b (5 mg/kg) could alter liver functions in 4T1 tumor-bearing animals. There were no alterations in the level of serum alanine aminotransferase (ALT) in all groups. However, the serum aspartate transaminase (AST) level was significantly elevated in the carboplatin (5 mg/kg) group but not in the LyeTx I-b-treated group (Figure 9). Therefore, our data suggest that LyeTx I-b treatment was not significantly different from the control and did not induce hepatotoxicity, similar to carboplatin, in the 4T1 murine mammary carcinoma.

### 2.9. LyeTx I-b and Carboplatin Reduce the Total and Differential Leukocyte Counts

Immunological analyses are relevant to evaluate the immunomodulatory effects of chemotherapeutic agents. Indeed, the aggressive 4T1 tumor cells growth and migration to the spleen and liver resulted in increased hematopoietic activity and, therefore, elevated circulation of white blood cells [44]. Post-inoculation of 4T1 cells into Balb/c mice increased by three-fold the total leukocyte counts at day 14 (i.e., the beginning of the metastasis process, which corresponds to multiplying numbers of granulocytes, mainly neutrophil and eosinophil counts, due to changes in immunity during metastasis process) [45].

**Figure 10:**
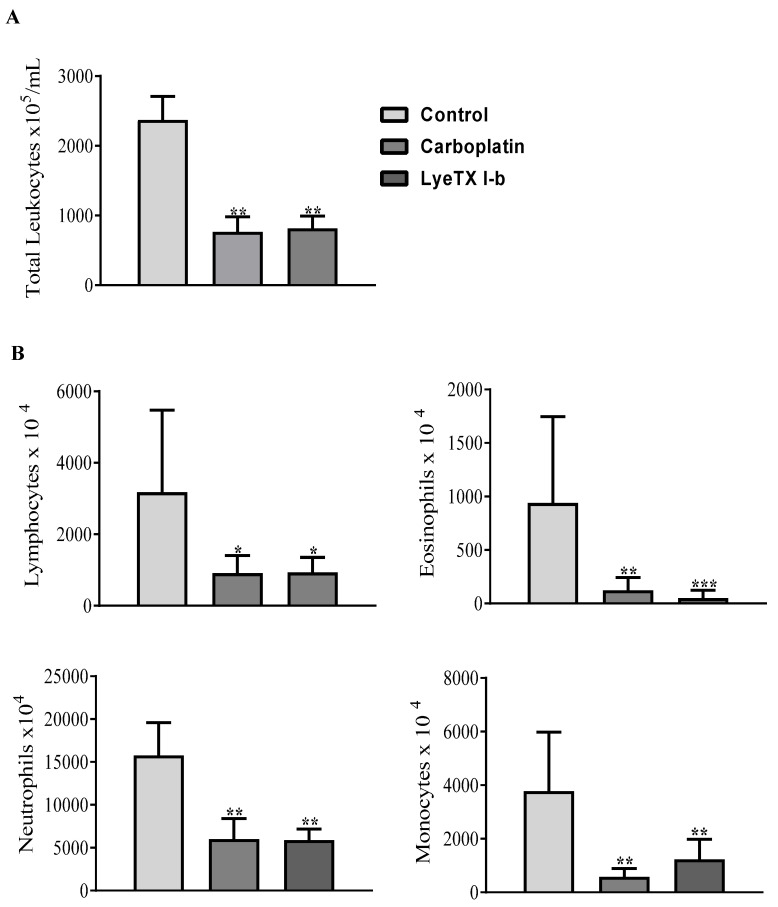
Leukogram of peripheral blood counts in 4T1 tumor-bearing mice. The control received saline, and the other groups received carboplatin or LyeTx I-b (5 mg/kg), five doses every 72 h. (**A**) Total leukocytes count × 10^5^. (**B**) Differential leukocytes count × 10^4^, lymphocytes, eosinophils, neutrophils, and monocytes. LyeTx I-b and carboplatin induced leukopenia in treated groups. Data are presented as the mean ± SD, * *p* < 0.05; ** *p* < 0.01; *** *p* < 0.001 compared to the control group, *n* = 6 animals.

After the collection of blood samples, the count of total and differential leukocytes (neutrophils, lymphocytes, monocytes, and eosinophils) was performed. We found that both drugs, carboplatin and LyeTx I-b, significantly decreased the total and differential number of peripheral blood leukocytes compared to the control group (Figure 10A). Currently, the attractive target in breast cancer murine models is the depletion of tumor-infiltrating cells, such as neutrophils and monocytes, which differentiate into metastasis-associated macrophages [46]. Our results showed that neutrophil, eosinophil, and monocyte counts were dramatically reduced after the treatment with LyeTx I-b and carboplatin (Figure 10B).

### 2.10. LyeTx I-b Alters the Numbers of Rolling and Adhering Leukocytes

**Figure 11:**
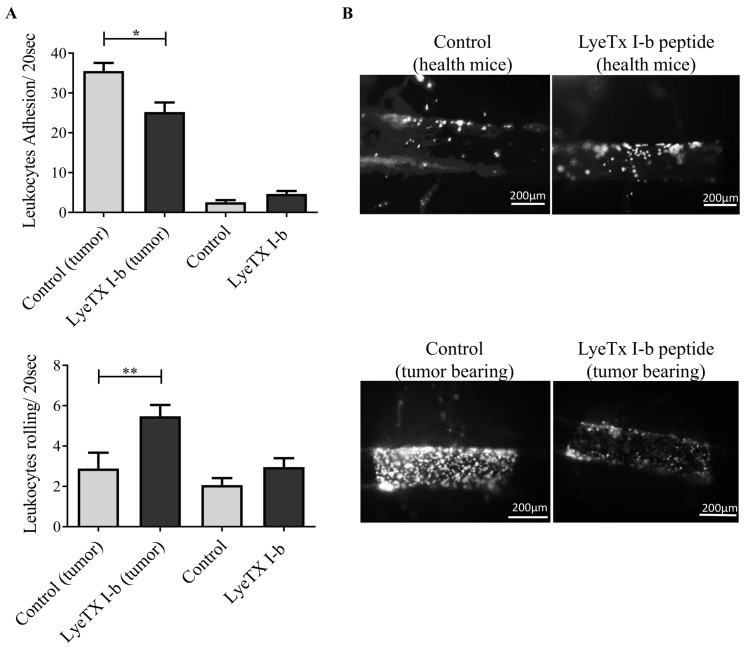
Evaluation of in vivo leukocyte recruitment by intravital microscopy, on day22 post-inoculation of 4T1 tumor cells. (**A**) Number of rolling and adhering leukocytes in health and tumor-bearing mice. Both control groups received 100 µL of saline, and treated groups received 5 mg/kg of LyeTx I-b dissolved in saline subcutaneously (5 doses/72 h apart). (**B**) Representative images of blood vessels of the spinal cord show the impact of LyeTx I-b treatment on leukocyte-endothelial interactions; higher numbers of adherent leukocytes in tumor-bearing control compared to the treated group were observed. Statistical analysis: Data are presented as the mean ± SD, * *p* < 0.05; ** *p* < 0.01, *n* = 6 animals. One-way ANOVA followed by Tukey’s multiple comparisons test.

The main goal of using an intravital microscope is to assess the impact of LyeTx I-b on leukocyte-endothelial interaction during the metastatic stage in vivo by quantifying leukocytes rolling and adhesion in the spinal cord microcirculation. To achieve this goal, we first determined central inflammation induced by 4T1 mammary tumor in the metastatic stage by measuring leukocyte extravasation, a mechanism associated with blood-brain-barrier breakdown [47]. Our results emphasized the remarkable increase of spinal cord rolling leukocytes after the treatment with LyeTx I-b compared to the control group. Moreover, we also observed a reduction in spinal cord leukocyte adhesion in 4T1 tumor-bearing mice treated with LyeTx I-b (Figure 11).

### 2.11. LyeTx I-b Decreases the Level of Cytokines in the Tumor Microenvironment

**Figure 12:**
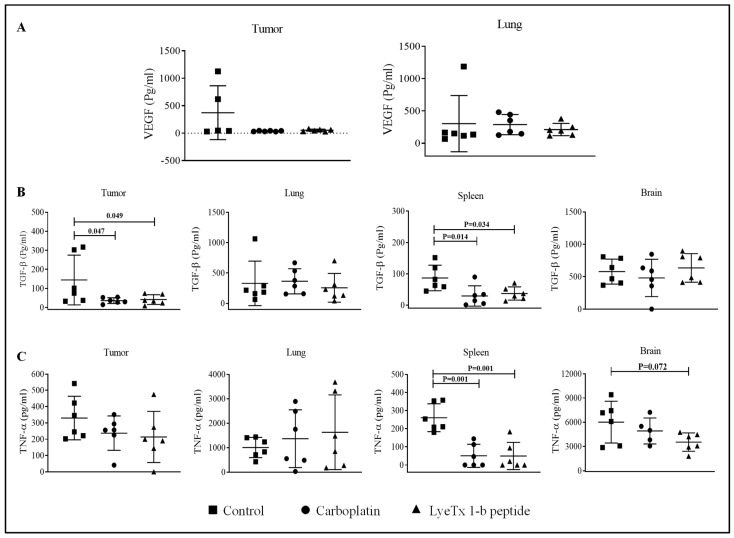
Cytokines levels in the tumor LyeTx I-b attenuated VEGF, TGF-β, and TNF-α levels in the tumor microenvironment. Individual values of mice (pg/mL) are presented in a dot plot with an average of each group, *n* = 6. (**A**) Data correspond to VEGF differences found in primary tumor and lung. (**B**,**C**) TGF-β and TNF-α values in the primary tumor, brain, spleen, and lung. Statistical variation among groups was carried out in 6 animals of each group in duplicate using One-way ANOVA, *p* < 0.05.

The evaluation of the level of cytokines and tumor growth factors, such as VEGF, TGF-β, and TNF-α, in the tumor microenvironment is important to understand their roles in tumor growth regulation, tumorigenesis, and metastasis. We found that the groups treated with LyeTx I-b and carboplatin did not exhibit statistical significance in VEGF concentration (pg/mL) of primary tumor and lung tissues (Figure 12A), while these compounds induced a significant reduction in TGF-β expression level in primary tumor and spleen compared to the control (Figure 12B). Furthermore, no significant difference in TNF-α expression in primary tumor was observed, whereas a significant reduction in the spleen was evidenced in LyeTx I-b and carboplatin groups (Figure 12C).

**Figure 13:**
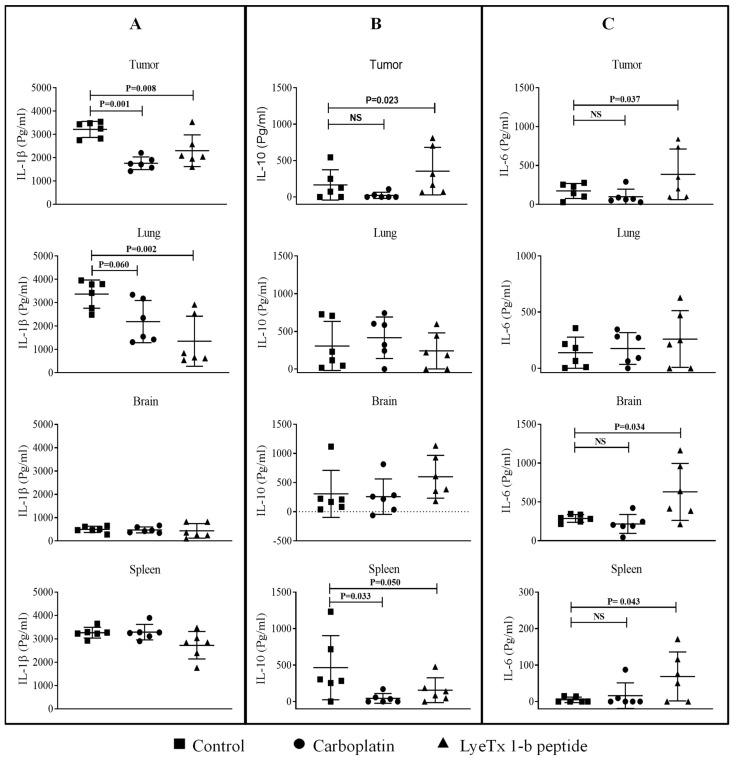
LyeTx I-b changed levels of IL-1β, IL-6, and IL-10 expression in the tumor microenvironment. Individual values of mice (pg/mL) are presented in a dot plot with an average of each group, *n* = 6. (**A**) Data correspond to IL-1β found in the primary tumor, brain, spleen, and lung. (**B**,**C**) IL-10 and IL-6 levels, respectively. Statistical variation among groups was carried out in 6 animals of each group in duplicate using One-way ANOVA, *p* < 0.05. NS means not statistically significant.

Inflammatory cytokines have a dual role in the tumor microenvironment based on the plasticity of immune cells. Our results showed that LyeTx I-b and carboplatin treatments significantly suppressed proinflammatory IL-1β expression in the lung and primary tumor (Figure 13A). On the other hand, the anti-inflammatory IL-10 expression was increased only in the primary tumor treated with LyeTx I-b (Figure 13B). By contrast, the proinflammatory IL-6 was significantly elevated in the brain and spleen in the group treated with LyeTx I-b (Figure 13C).

## 3. Discussion

In this study, we evaluated the link between the in vitro cytotoxicity and in vivo antitumor activity of LyeTx I-b. As previously mentioned, to investigate the in vivo anticancer effect of the LyeTx I-b peptide, the 4T1 murine breast cancer model was selected based on the importance of new drugs for triple-negative breast cancer treatment. Viability data demonstrated that this peptide was active against murine triple-negative breast cancer lineage at low concentrations (<10 µM). The IC_50_ values of LyeTx I-b obtained against 4T1, MCF-7, and MDA-MB-231 were at a lower micromolar range (6.5 µM, 7.34 µM, and 5.77 µM, respectively), in comparison to carboplatin (>100 µM). The data obtained for carboplatin in our experimental conditions are in agreement with Łakomska and colleagues. These authors tested the effects of carboplatin on 4T1 and T47D (human ductal breast carcinoma) using the MTT assay and found that the IC_50_ values for these cells were 84 µM and 134 µM, respectively [48]. The MTT method estimates total cell survival, but it does not determine the surviving fraction of clonogenic cells to predict in vivo antitumor activity. One of the goals of anticancer drugs is to induce cell death or the loss of clonogenic cells [42].

The clonogenic assay is a predictive and informative methodology to determine the sensitivity of anticancer agents to radiation [49,50]. We performed this assay to predict the potential tumoricidal effect of LyeTx I-bin vitro and decide to go further to in vivo experiments. LyeTx I-b significantly inhibited the clonogenic survival of 4T1 cells (Figure 1) at IC_50_ (6.5 µM), suggesting anticancer potential in vivo. Complete inhibition of 4T1 clonogenic survival cells was observed at IC_80_ (25 µM). Several studies reported that cationic peptides reduce the survival of colonies of breast cancer cells by blocking the transformation of the single cell into colonies [51]. Indeed, the reduction in viability and clonogenic survival of 4T1 cells after the treatment with LyeTx I-b seems to be associated with apoptosis induction, which is a desired effect of apotential anticancer candidate. These data were supported by the increase in sub-diploid DNA content in cells treated with the peptide or carboplatin (Figure 2A) and by the labeling with annexin-V, observed by flow cytometry analysis (Figure 2B). 

Apoptosis is one of the mechanisms responsible for the anticancer effect of drugs and peptides [52]. Necrosis and apoptosis induce an increase in DNA sub-diploid. Thus, the FITC-conjugated Annexin V/PI protocol was conducted to differentiate these two mechanisms. 4T1 cells treated with LyeTx I-b 4T1 presented cell population stained by Annexin V and Annexin/PI compared with control cells. In contrast, PI staining of necrotic cells was negligible in both control and treated cell groups. These data suggested that LyeTx I-b induces cell death by apoptosis of 4T1 cells. These results were different from those we obtained with LyeTx I-b against U-87 MG glioblastoma cells, which the primary death mechanism seems to be through necroptosis [41]. It suggests that LyeTx I-b may have different mechanisms of action depending on the type of tumor. The antimicrobial peptide temporin-1CEa induced apoptosis in MCF-7 and MDA-MB-231 breast cell lines through phosphatidylserine externalization of the cell surface, which was confirmed by FITC-annexin V positive [53]. Similarly, the cationic peptideLL-37 induced apoptosis in Jurkat T leukemia cells characterized by DNA sub-diploid and internal cell surface reflection, which resulted in the increase of Annexin V staining [54].

Nowadays, chemotherapy based on biopharmaceutics, such as peptides, became one of the most promising strategies to treat metastatic breast due to their efficiency and mild adverse effects [18,55]. Besides that, therapeutic peptides have high activity and are easily synthesized and modified [55]. The systemic administration of LyeTx I-b did not cause signs of toxicity, and no significant alterations were observed in histopathological and hematological analyses. These results are compatible with the findings for the PFR peptide, which inhibited tumor growth without toxic side effects [56].

The intratumoral injection of LyeTx I-b suppressed 52% of tumor growth and promoted a 61% reduction of pulmonary metastatic foci in comparison to the control group. To confirm the death of primary tumor, the qualitative histopathological analysis of the tumor section was performed. The data showed a large area of necrosis and pyknosis in the treated group characterized by the occurrence of nuclei irreversible chromatin condensation and fragmentation. These effects have also been observed after the treatment with other peptides. For example, intratumoral administration of Cypep-1, a synthetic cationic peptide, induced tumor regression in the 4T1 murine model and necrosis in a large area of the treated primary tumor [57]. Moreover, local injection of L-K6, a nine amino acid-residue peptide fragment derived from human lactoferricin, induced nuclear fragmentation in the primary tumor of the MCF-7 breast cancer xenograft model [58].

Subcutaneous injection is one of the most important routes for biotherapeutics administration. It ensures the high bioavailability of compounds with high molecular weights [59] and delivers therapeutic peptides to the systemic circulation by the lymphatic system and blood capillaries [60]. LyeTx I-b was administrated subcutaneously every 72 h over 14 days, and our findings showed a reduction of 58% in the tumor growth compared to the tumor-bearing control. Subcutaneous injection of crotamine, a peptide obtained from a snake venom of *Crotalus durissus terrificus* viper, significantly delayed tumor growth in the B16-F10 melanoma model [61].

The proportions of rolling and adhering leukocytes always increase under tumorigenesis and metastasis illness conditions [62]. Tumor cells in metastatic lesions utilize adhering leukocytes for indirect extravasation into endothelium by recruiting leukocytes [63]. The P-selectin expression on tumor cells correlated with cancer metastasis [64]. Rolling leukocytes are also mediated by the L-selectin adhesion molecule, which is a key regulator of naive T-lymphocytes trafficking [65]. We speculated that LyeTx I-b might modulate integrin molecules on the surface of leukocytes, which are responsible for the interaction with endothelium cells. Another mechanism that may decrease leukocyte adhesion is the high and fast migration of leukocytes to the surrounding parenchyma or distant organs. To elucidate potential mechanisms of action of LyeTx I-b, additional experiments will be necessary.

Due to the aggressive tumor growth of 4T1 murine mammary carcinoma, previous studies reported that 4T1 is a highly immunogenic model characterized by leukocytosis due to granulocytic hyperplasia of bone marrow [27]. All solid tumors, including breast tumors, contain large numbers of leukocytes that promote tumor migration and splenomegaly by tumor-infiltrating monocytes and neutrophils. Thus, targeting the spleen during cancer treatment is an urgent need for controlling tumor migration [66]. Some studies reported that chemotherapeutic agents caused immunosuppression by killing immune cells, such as cytotoxic T-lymphocyte–associated antigen 4 and monocytes or macrophages infiltrated tumor that promotes cancer metastasis [67,68]. Conventional chemotherapeutic agents induce cytotoxic effects and prevent tumor development through the depletion of tumor-infiltrating immune cells [69]. LyeTx I-b presented an immunomodulatory effect by attenuating counts of monocytes, lymphocytes, neutrophils, and eosinophils in the 4T1 metastatic model.

Immune cells and their secretions, such as cytokine and chemokine, interfere with the tumor microenvironment and, depending on the type of inflammatory mediators produced by neutrophils, lymphocytes, and monocytes, give rise to tumor growth or regression [70]. Overexpression of TGF-β, VEGF, and TNF-α have been detected in murine 4T1 metastatic model and breast cancer patients, leading to a reduction in the incidence of metastases and increasing the patient’s survival rate [71]. The proportional correlation between high levels of TGF-β and VEGF and the early appearance of metastatic lesions was reported. The blockade of TGF-β and VEGF resulted in improved efficiency of chemotherapeutic agents in 4T1 mammary tumor model and breast cancer patients through the recruitment and normalization of extracellular matrix, particularly collagen-coated matrices [72,73]. The pleiotropic cytokine tumor necrosis factor (TNF) is widely accepted as a central player in the multi-faceted tumor microenvironment, showing a dual role as tumor-promoting or tumor-suppressing, depending on the binding to its receptors TNF 1 (TNFR1) and TNF 2 (TNFR2). TNF promotes apoptosis via binding to TNFR1 but plays a prosurvival function via TNFR2. TNFR2 is expressed in some tumor cells and suppressive immune cells, including regulatory T cells and myeloid-derived suppressor cells. In this context, although TNF-α is utilized as a cancer immunotherapeutic agent, recently, several studies emphasized its ability to promote tumor progression and metastasis by the upregulation of the VEGF receptor. In addition, TNF-α facilitates the proliferation of cancer cells in the immune microenvironment [74,75]. Our results demonstrated that LyeTx I-b inhibited the expression of TGF-β in primary tumor and spleen. In addition, carboplatin and LyeTx I-b decreased the production of TNF-α in the spleen, which can accumulate tumor-promoting immune cells responsible for the metastatic process.

A previous clinical study showed the dual role of cytokines in the breast cancer microenvironment during tumor progression. VEGF, TNF-α, IL-1β, and IL-6 expression levels documented in breast cancer patients were increased in the biopsies tissues compared to normal tissues [76]. IL-1β expression plays an essential role in tumor progression and metastasis. It is also considered bad prognoses in several cancers, such as breast, lung, prostate, neck, and head. Moreover, inhibition of the IL-1 receptor resulted in tumor regression of solid malignancies [77]. Similarly, blocking of the IL-1 receptor by Anakinra antagonist exhibited a significant reduction in breast cancer progression and bone metastasis in MCF-7 and MDA-MB-231 breast cancer models [78]. In contrast, over expression of IL-10 protected from carcinogenic and enhanced tumor immune surveillance through the activation of intratumoral antigen-presenting molecules, cytotoxic CD8+, and IFN-γ. Subsequently, IL-10 can inhibit tumor migration and progression, especially in the early stage of breast cancer [79,80]. 

It is worth mentioning that the peptide LyeTx I-b significantly decreased the expression of IL-1β in tumor and lung tissues, with an increase in the levels ofIL-10 anti-inflammatory cytokines in primary tumor, as observed with other AMPs [81]. The results were consistent with a previous study that indicated that LyeTx I-b decreased IL-1β level in the joint of septic arthritis mice model [37]. Interestingly, the production of IL-6 increased in the tumor, spleen, and brain (Figure 13C). Although tumor inoculation of the 4T1 cell line generated greater levels of TNF-α, IL-1β, IL-6 in the spleen, serum, primary tumor, and liver, the IL-1β cytokine increased only in the cortex and hippocampus [46]. Several studies have shown that chemotherapy can activate toll-like receptors 4 (TLR4), which exhibited an increase in proinflammatory IL-6 cytokine in blood and organs distant from the primary tumor in animal models and breast cancer patients [82]. Subsequently, widely used cytotoxic drugs, such as 5-fluorouracil, doxorubicin, and paclitaxel, may cause fatigue, cachexia and facilitate tumor progression in cancer survivors [83,84]. 

AMPs are a part of innate host defense with dual roles, antimicrobial and immunomodulation [85]. Interestingly, AMPs could control immune response through binding with cytokines receptor in monocytes, dendrites, and lymphocytes [85,86]. It is well described that AMPs triggered breast malignant tumors through various mechanisms like immunomodulation, cell cycle arrest, inhibition of intracellular targets, and cell membrane disruption [25,87]. Based on these previous results with other cationic, but structurally different AMPs, we can speculate that LyeTx-I b could interact with chemokines receptors inducing immunomodulatory effect through reduction of immune cells in the bloodstream and adhesion leukocytes in the spinal cord, followed by downregulation of TGF- β, IL-1β, and TNF-α in the primary tumor. In addition, it seems upregulate IL-6, due to the cytotoxic effect. Also, it is important to mention that LyeTx-I b might attenuate the infiltration of cytotoxic lymphocytes cells into tumor lesions which are responsible for tumor progression to metastatic stage. We suggest that possibly, LyeTx-I b might alter signaling cascades in the tumor microenvironment. However, other studies are being carried out to better understand the action mechanism of this peptide on breast tumor.

## 4. Materials and Methods

### 4.1. Cell Lines

The murine triple-negative 4T1 mammary adenocarcinoma cells were kindly provided by Dr Miriam Teresa Paz Lopes, Departamento de Farmacologia, Universidade Federal de Minas Gerais. In addition, The MCF-7 (estrogen-positive and progesterone receptor-positive) and MDA-MB-231 (triple-negative) human breast carcinoma lineages were kindly provided by Dr. Marcel Leist (University of Konstanz/Germany). These lineages were maintained in the logarithmic phase of growth in high glucose DMEM (Dulbecco’s Modified Eagle Medium) culture medium supplemented with 10% fetal bovine serum, and incubated at 37 °C in a 5% CO_2_ atmosphere and 1% antibiotic solution (100 IU/mL penicillin and 100 µg/mL streptomycin (GIBCO BRL, Grand Island, NY, USA). 

### 4.2. Evaluation of Cell Viability Using the MTT Assay

The MTT (3-(4,5-dimethylthiazol-2-yl)-2,5-diphenyltetrazolium bromide) assay is a standard colorimetric assay in which mitochondrial activity is measured based on the metabolic reduction of MTT, allowing the evaluation of cell proliferation and viability. Different lineages were seeded at a density of 10,000 cells/well (96-well plate) and treated for 48 h with the peptide LyeTxI-b (100–0.75 µM) or Carboplatin (Sigma^®^ Co., Brazil) (300–1.5 µM), followed by the addition of 2.5 mg/mL MTT (Sigma^®^ Co., St. Louis, MO, USA) (30 µL per well). The plates were incubated for 4 h at 37 °C in 5% CO_2_; for obtaining formazan crystals, the supernatant was removed, and 200 µL of HCL isopropanol (0.04 M) was added. After gently stringing for 5 min at room temperature, the absorbance of the solubilized MTT formazan product was measured at 595 nm (Spectramax-Molecular Devices^®^, San Jose, CA, USA) [88]. 

### 4.3. Cell Cycle Analysis

The quantification of the sub-diploid DNA content was performed using flow cytometry, as previously described [89]. The 4T1 cells were seeded in 24-well plates at a confluence of 100,000 cells/well and incubated overnight at 37 °C for stabilization. Then, 4T1 cells were incubated with 6.5 µM of LyeTxI-b for 48 h. After the treatment, the cells were transferred to 1 mL tubes and centrifuged at 10,000 rpm for 5 min in a microcentrifuge (Denver Instrument Company, Denver, CO, USA). The supernatant was discarded, and 300 µL of a Hypotonic Fluorochromic Solution (HFS) containing 50 µg/mL of Propidium Iodide—PI (Sigma, Saint Louis, MO, USA) and 0.1 % of Triton X-100 (Sigma, Saint Louis, MO, USA) in 0.1 % sodium citrate (Sigma, Saint Louis, MO, USA) was added. The plates were incubated for 4 h at 4 °C. After incubation, the samples were submitted to flow cytometry FACS (Fluorescence Activated Cell Sorting) analysis. The FL2 voltage was adjusted so that the G0/G1 and G2/M phases formed peaks of values of 200 and 400, respectively, in FL2-H. The values of FSC-H, SSC-H, FL2-Hand FL2-W were acquired for histograms, and statistical analysis was conducted using the FlowJo 7.6.4^®^ (Tree Star Inc., Ashland, OR, USA) [90]. 

### 4.4. Quantitation of Cell Death Using Annexin V and PI

The detection of apoptosis and necrosis was measured by Annexin V Binding and Propidium Iodide uptake, following the supplier’s recommendations. Briefly, 4T1 cells were maintained in the DMEM culture medium, supplemented with 10% fetal bovine serum. The cells were harvested and seeded in 24-well plates at a confluence of 200,000 cells/mL and incubated overnight at 37 °C for stabilization. Then, 4T1 cells were incubated with 6.5 µM LyeTxI-b for 24 h. The cells were then transferred to 1 mL tubes and centrifuged at 10,000 rpm for 5 min in a microcentrifuge (Denver Instrument Company, Denver, CO, USA). The supernatant was discarded and treated and untreated cells were stained with 2.5 µL of Annexin V and Propidium Iodide (BD Biosciences) for 15 min in the dark at room temperature. Finally, the samples were submitted to the FACS Calibur flow cytometry analysis [57]. The values of FSC-H, SSC-H, were acquired for histograms, and statistical analysis was conducted using FlowJo 7.6.4^®^ (Tree Star Inc., Ashland, OR, USA).

### 4.5. Evaluation of the Clonogenic Survival of 4T1 Cells after the Treatment with LyeTx I-b

The 4T1 cells were maintained in DMEM medium supplemented with 10% FSB for 24 h in 5% CO_2_. After the cell’s trypsinization process, they were resuspended in sufficient media volume to neutralize the trypsin enzyme. Additionally, 4T1 cells were quantified, and 300 cells were immediately re-plated in six-well tissue culture plates for 24 h. Then, the cells were treated with 6.5 µM (IC_50_) and 25 µM (IC_80_) of LyeTxI-b for 12 h. After the incubation period, the medium was removed, the cells were carefully rinsed with PBS, fresh culture medium was added, and the cells kept growing for 10 days. Plates were fixed with ethanol 70% for 40 min and stained with 0.5% crystal violet for 15 min. The ethanol and crystal violet mixtures were carefully removed, and the plates were rinsed with tap water [42]. The fraction of plating efficiency surviving was calculated.

### 4.6. Animals and Procedures

Females Balb/c mice (9–10 weeks old) were obtained from the animal facility of the Universidade Federal de Minas Gerais (UFMG). The animals were maintained under temperature-controlled conditions with an artificial 12-h light/dark cycle and were provided with standard chow and water *ad libitum.* Efforts were made to avoid any unnecessary distress to the animals. Animal procedures were approved and carried out in accordance with the Animal Ethics Committee of UFMG, under protocol number 39/2018.

### 4.7. Sub-Acute Toxicity of LyeTx I-b Peptide In Vivo

The peptide LyeTx I-b was acquired from GL Biochem (Shanghai, China) with a purity of 99%, which was confirmed by chromatographic and spectrometric analyses.

Balb/c mice were randomly assigned to two groups: control (*n* = 6) and treated (*n* = 6). Animals were intraperitoneal injected with 100 µL of saline or peptide (5 mg/kg/day) (seven doses, 48 h apart) and were monitored for 15 days. Body weight measurements were monitored three times a week to examine the toxicity of LyeTx I-b. Eighteen females BALB/c mice were anesthetized with a mixture of ketamine (150 mg/kg) and xylazine (10 mg/kg) and perfused through the heart with 10% formalin. The tumor, spleen, lung, brain, kidney, and liver were quickly removed and fixed in 10% formalin for 48 h until subsequent histological analysis. 

### 4.8. Tumor Inoculation and Drug Treatment

4T1 cells were detached from the T75 flask surface by 3 mL trypsin solution (0.05%), suspended in sufficient volume of DMEM medium to neutralize the trypsin, and then centrifuged at 300× *g* for 5 min. After counting, the cells were resuspended in PBS (1 × 10^6^/100 µL). To obtain the solid tumor, 100 µL were injected subcutaneously into the left flank of all animals [91]. 

### 4.9. Intratumoral Drug Administration

Thirty-six mice were randomly divided into six groups. On day 10 post-inoculation, three groups received an intratumoral injection of saline (100 µL), carboplatin, or LyeTx I-b, respectively (five doses of 5 mg/kg, 72 h apart). On the fifth day, the other three groups received the same treatment subcutaneously. The body weight and tumor volume were monitored every three days, and signs of distress and pain were monitored daily [92]. The measurement of tumor volume was calculated using the following equation: tumor volume (mm^3^) = length (mm) × width^2^ (mm)/2 [93]. The blood samples were collected by cardiac puncture, and tissues, such as tumors, spleens, lung, brain, kidney, and liver were harvested and fixed in 10% formalin for histological analysis.

### 4.10. Subcutaneous Cells and Drug Administration

Eighteen females BALB/c mice were injected subcutaneously (s.c.), with 4T1 cells into the left flank after five days when the tumor volume reached approximately 0.25 cm^3^. Then, all animals were randomly assigned to three main groups: saline (s.c.) (*n* = 6), carboplatin (s.c., 5 mg/kg) (*n* = 6), and LyeTxI-b (s.c.; 5 mg/kg) (*n* = 6). All animals received the treatment five subsequent times every 72 h. The body weight and tumor volume were monitored every three days. Signs of distress and pain were monitored daily.

### 4.11. Histological Analysis

According to tissue preparation for conventional histology protocol, after fixation, transverse sections were obtained, embedded in paraffin, cut into 4 µm sections, and stained with hematoxylin and eosin (H&E). The images were captured using light microscopy (Olympus, Miami, FL, USA) with a plan apochromatic objective 20X [94]. All images were digitized by Spot Insight Color (SPOT^®^ version 3.4.5.) (Diagnostic Instruments Inc., Sterling Heights, MI, USA). A micro camera (Olympus Microscope, BX-40, Miami, FL, USA) mounted on the microscopy projected the images to a monitor, which were analyzed by software. In order to evaluate the metastatic nodules, morphometric image analysis was performed using a 40× objective [95].

### 4.12. Evaluation of Liver Integrity

Hepatic damage leads to an increase in the plasma aspartate aminotransferase (AST) and alanine aminotransferase (ALT) enzymes activities. The quantification of the activity of the AST and ALT was performed as previously described [96]. The absorbance reading (at 540 nm) was done by using a spectrophotometer (BioTek, Instruments, Winooski, VT, USA). The data filled a linear regression allowing to calculate ALT and AST activities, which were expressed as international units per liter (IU/L).

### 4.13. Complete Blood Count

Mice were euthanized, blood samples were collected in a heparinized tube, and 100 µL of whole blood were submitted to ABC Vet automatic blood analyzer for counting of red blood cells (RBC), hematocrit, hemoglobin, platelets, white blood cells (WBC), RBC parameters, mean cell volume (MCV), mean corpuscular hemoglobin (MCH), and mean corpuscular hemoglobin concentration (MCHC).

### 4.14. Manual Quantification of Differential Leukocytes

Whole blood specimens were collected in 0.5 M sodium EDTA. Blood smear film on a microscopic glass slide was obtained by quickly and smoothly spreading one blood drop (30 µL) close to the end of the slide. Then, the peripheral blood film was stained with May-Grünwald’s eosin methylene blue and Giemsa’s azure eosin methylene blue pH 6.4–7. Upon the staining of differential leukocyte populations, the cells were counted using a light microscope; 100 or 200 cells were manually counted.

### 4.15. Detection and Quantitation of Cytokines and Growth Factors

Cytokines and growth factors in the primary tumor, lung, spleen, and brain were determined using ELISA. Tissues were removed and preserved at −80 °C until analysis. The amount of 100 mg from each tissue was homogenized in a solution (1.0 mL) containing 0.05% tween 20, 0.5% BSA, 0.0017% PMSF, 0.005% benzethonium chloride, 0.0037% EDTA and aprotinin 2 µL/100 mL, diluted in phosphate buffer saline (pH 7.4) and then, centrifuged at 10,000 rpm at 4 °C for 10 min. Then, 50 µL of supernatant from each tissue were used to measure growth factor and cytokines by using murine antibodies of VEGF, TGF-β, TNF-α, IL-1β, IL-10, and IL-6, DuoSet ELISA kit (R&D Systems, Minneapolis, MN, USA) according to manufacturer’s protocol to detect antigen-containing sample to the plate [97].

### 4.16. Intravital Microscopy

Intravital microscopy was used to evaluate in vivo leukocyte recruitment (rolling and adhesion), as previously described [98]. Briefly, after the induction of 4T1 mammary carcinoma model, 24 females BALB/c mice (9–10 weeks old) were randomly assigned to four groups: control (saline, s.c.), 4T1 tumor-bearing treated with saline, 4T1tumor-bearing treated with LyeTxI-b (5 mg/kg), and control treated with only LyeTxI-b (5 mg/kg). All animals received treatments for five subsequent times every 72 h. After 21 days of tumor inoculation, animals were anesthetized, and microvasculature from the spinal cord (bylaminectomy), flank (control animals), and tumor were exposed to analyze the in vivo leukocyte recruitment by intravital microscopy. Then, retro-orbital injection of rhodamine 6G (1 mg/mL) was performed for labeling platelets and leukocytes. After microcirculation exposure, animals were transferred to the microscope stage and maintained at 37 °C using a heating pad (Fine Sciences Tools Inc., Vancouver, BC, Canada). To assess the leukocyte-recruitment interactions, fluorescent leukocytes were visualized under fluorescence microscopy Zeiss Imager M.2 (20× long-distance objective lens; Gottingen, Germany) equipped with a fluorescent light source (epi-illumination at 510–560 nm, using 590 nm emission filter). Rolling leukocytes were defined as white cells moving at a lower speed than erythrocytes. Cells were considered adherent when they remained stationary for at least 20 s, and the results were expressed as leukocytes number/20 s [42].

### 4.17. Statistical Analyses

Statistical analyses were performed using Prism software version 7 (GraphPad Software, San Diego, CA, USA). All data were expressed as mean ± SD. One-way analysis of variance (ANOVA) was used to compare multiple groups. To compare only two groups, an unpaired *t*-test was performed. The significance level was established at *p* < 0.05. 

## 5. Conclusions

Altogether, these findings demonstrate that LyeTx I-b reduced tumor growth, with anti-metastatic and immunomodulatory effects in the 4T1 triple-negative breast cancer murine model. Therefore, all results suggest that this cationic antimicrobial peptide may be a potential immunomodulatory target for the development of breast cancer therapy in order to improve patient clinical outcomes.

## Figures and Tables

**Table 1:** IC_50_ values of LyeTxI-b and carboplatin against cancer cell lines.

Cell Line	LyeTx I-b IC_50_ (µM)	Carboplatin IC_50_ (µM)
4T1	6.5 ± 5.30	≥100
MCF-7	7.34 ± 3.09	≥100
MDA-MB-231	5.77 ± 0.83	69.20 ± 1.22
The IC_50_ values were calculated based on the linear regression of the dose-log response curves after 48 h exposure to the peptide or carboplatin, determined by the MTT and resazurin assays. The values represent the mean ± S.D. of at least 3 independent experiments. Representative concentration-response curves are displayed below.		
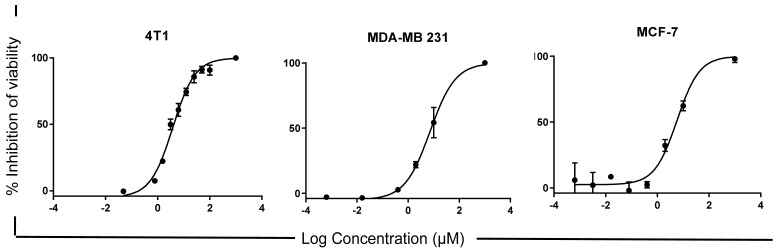		

**Table 2:** Hematological values of healthy BALB/C animals after systemic administration of 5 mg/kg LyeTx I-b (7 doses/48 h apart).

Hematological Parameter	Control	LyeTx I_b Peptide
RBC (×10^6^/mm^3^)	6.32 ± 0.667	7.46 ± 0.965
Hb (g/dL)	10.8 ± 2.66	12.9 ± 0.680
Hematocrit	38.9 ± 8.66	39.4 ± 2.56
MCV (fL)	61 ± 7.57	48 ± 4.50
MCH (Pg)	16.7 ± 0.458	16.0 ± 0.11
MCHC %	28.5 ± 3.06	31.6 ± 1.34
WBC (×10^3^/mm^3^)	2.3 ± 2.26	4.1 ± 0.70
Lymphocytes	91.7 ± 5.13	89.2 ± 4.03
Monocytes	4.3 ± 1.556	4.1 ± 1.553
Neutrophils	4 ± 3.59	6.7 ± 2.66
Platelets (×10^3^/mm^3^)	443 ± 73.6	278 ± 80.0 *

RBC: erythrocytes, Hb: hemoglobin, WBC: leukocytes, MCV: mean cell volume, MCH: mean corpuscular hemoglobin, and MCHC: mean corpuscular hemoglobin concentration. The results were expressed as mean ± SD. Unpaired *t*-test was performed. * (*p* = 0.004).

## Data Availability

Not applicable.
